# Supplementary figures and images for: Incomplete paralog compensation generates selective dependency on TRA2A in cancer
Source: PLoS Genet. 2025 May 14;21(5):e1011685. doi: 10.1371/journal.pgen.1011685 (PMC12077678; doi:10.1371/journal.pgen.1011685)

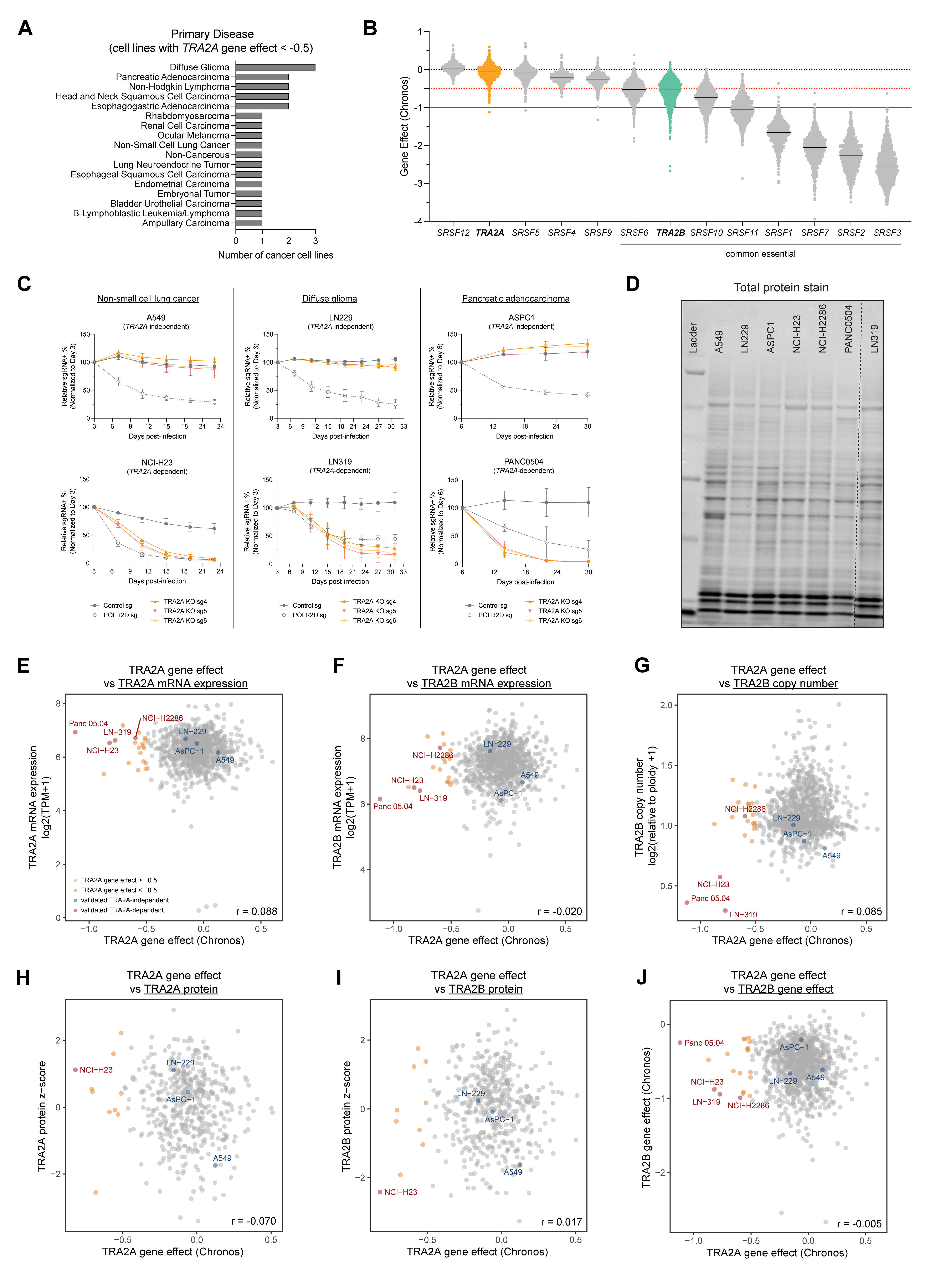

Supplement: S1 Fig — (A) Distribution of primary diseases among cancer cell lines from the Cancer Dependency Map (Public 24Q2 dataset) with a TRA2A gene effect score of less than -0.5. (B) Distribution of gene effect scores after knockout of indicated SR protein genes from the Cancer Dependency Map (Public 24Q2 dataset). Red lines mark gene effect scores of -0.5 (dotted) and -1.0 (solid). Also indicated are genes designated as ‘common essential’ by DepMap. (C) Competition-based proliferation assays performed in indicated TRA2A-independent and dependent Cas9 + cell lines after TRA2A KO, n = 3. (D) Representative image of total protein stain for normalization of TRA2A and TRA2B protein levels across cell lines (see Fig 1I). (E-J) Scatterplots from DepMap data of TRA2A gene effect scores vs (E) TRA2A mRNA expression (log2(TPM + 1)), (F) TRA2B mRNA expression (log2(TPM + 1)), (G) TRA2B copy number (log2(relative to ploidy+1)), (H) TRA2A protein expression (z-score), (I) TRA2B protein expression (z-score), or (J) TRA2B gene effect scores. Orange circles indicate cell lines with TRA2A gene effect scores < -0.5, red circles indicate validated TRA2A-dependent cell lines and blue circles indicate validated TRA2A-independent cell lines. (TIF) [file pgen.1011685.s001.tif]

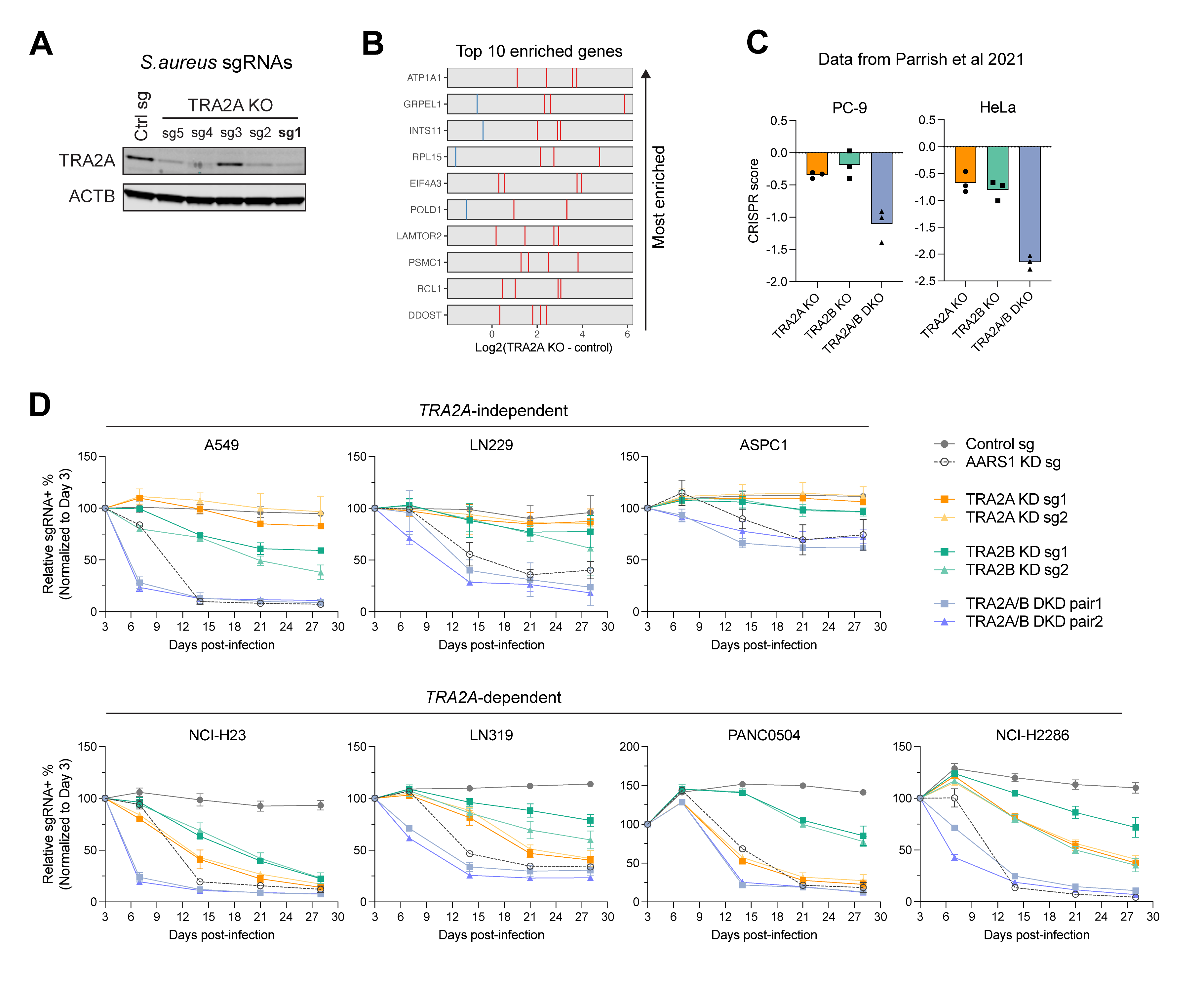

Supplement: S2 Fig — (A) Immunoblot showing depletion of TRA2A protein levels after S. aureus Cas9 targeting in NCI-H23 cells. ACTB was used as loading control. Bold indicates the guide used in the screen (sg1). (B) Log2 fold-change between TRA2A KO and control conditions of the individual guides targeting genes representing the 10 most enriched genes in A549 cells. (C) CRISPR scores calculated from single and double KO paralog screens in PC-9 and HeLa cells, obtained from Parrish et al. 2021 [27]. (D) Competition assays performed with single or dual KD of TRA2A and TRA2B in indicated Zim3-dCas9 + cell lines, n = 3. (TIF) [file pgen.1011685.s002.tif]

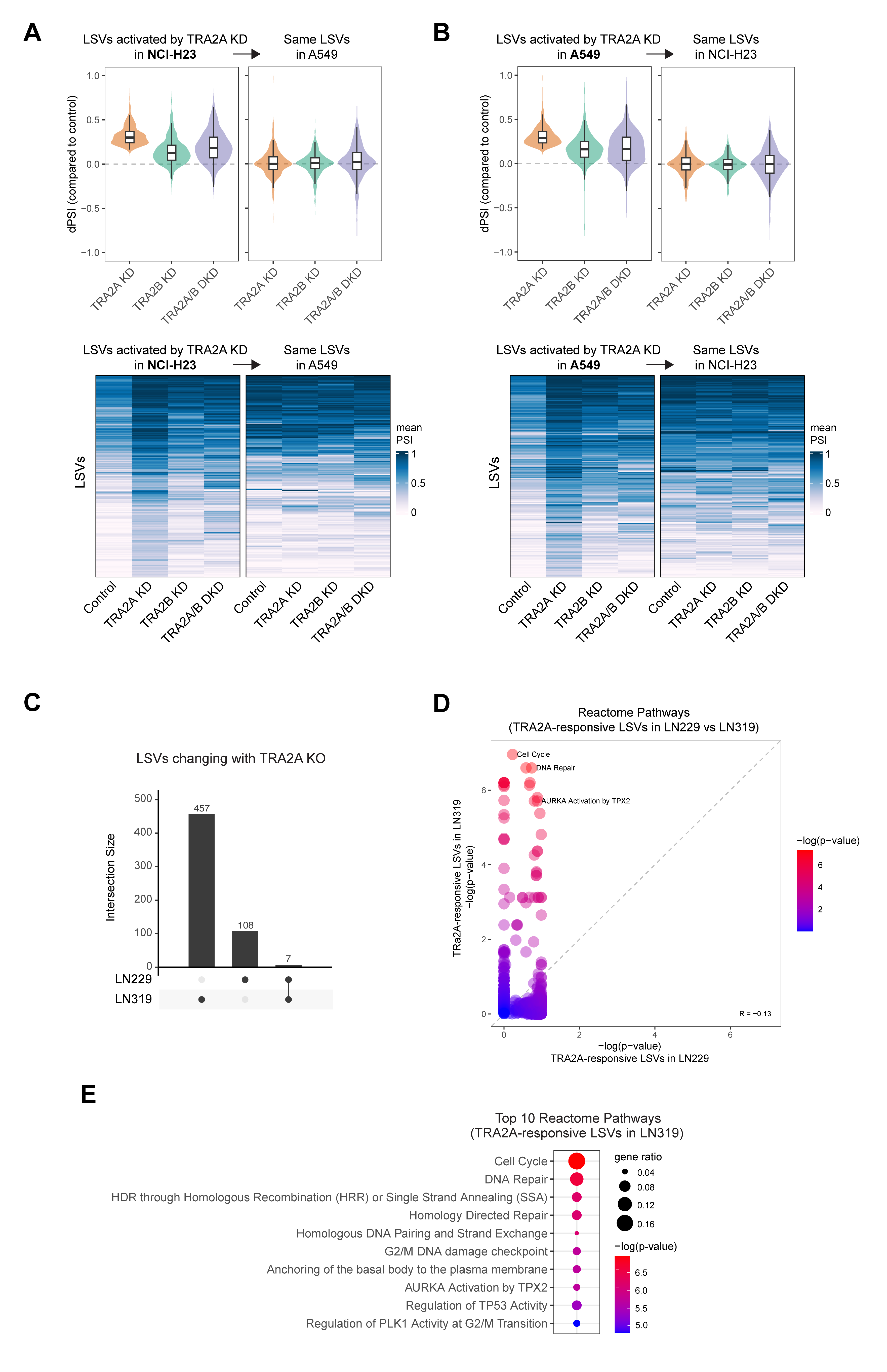

Supplement: S4 Fig — (A) (Top) Mean dPSI values of changing LSVs activated upon TRA2A KD in NCI-H23 cells, plotted for both NCI-H23 and A549 cells. (Bottom) Heatmap representation of the same LSVs showing their mean PSI values across all conditions in NCI-H23 and A549. Rows represent an individual LSV and heat color represents the mean PSI for a given LSV, n = 3. (B) (Top) Mean dPSI values of changing LSVs activated upon TRA2A KD in A549 cells, plotted for both A549 and NCI-H23 cells. (Bottom) Heatmap representation of the same LSVs from (Top) showing their mean PSI values across all conditions in A549 and NCI-H23. Rows represent an individual LSV and heat color represents the mean PSI for a given LSV, n = 3. (C) UpSet plot representing the overlap of changing LSVs upon TRA2A KO in LN229 and LN319. (D) Scatterplot showing enrichment of Reactome pathways for changing LSVs upon TRA2A KO in LN229 and LN319. Data plotted represents the -log(adjusted p-value) assigned to the Reactome term. (E) Top enriched Reactome pathways of changing LSVs responsive to TRA2A KO in LN319 cells. (TIF) [file pgen.1011685.s004.tif]

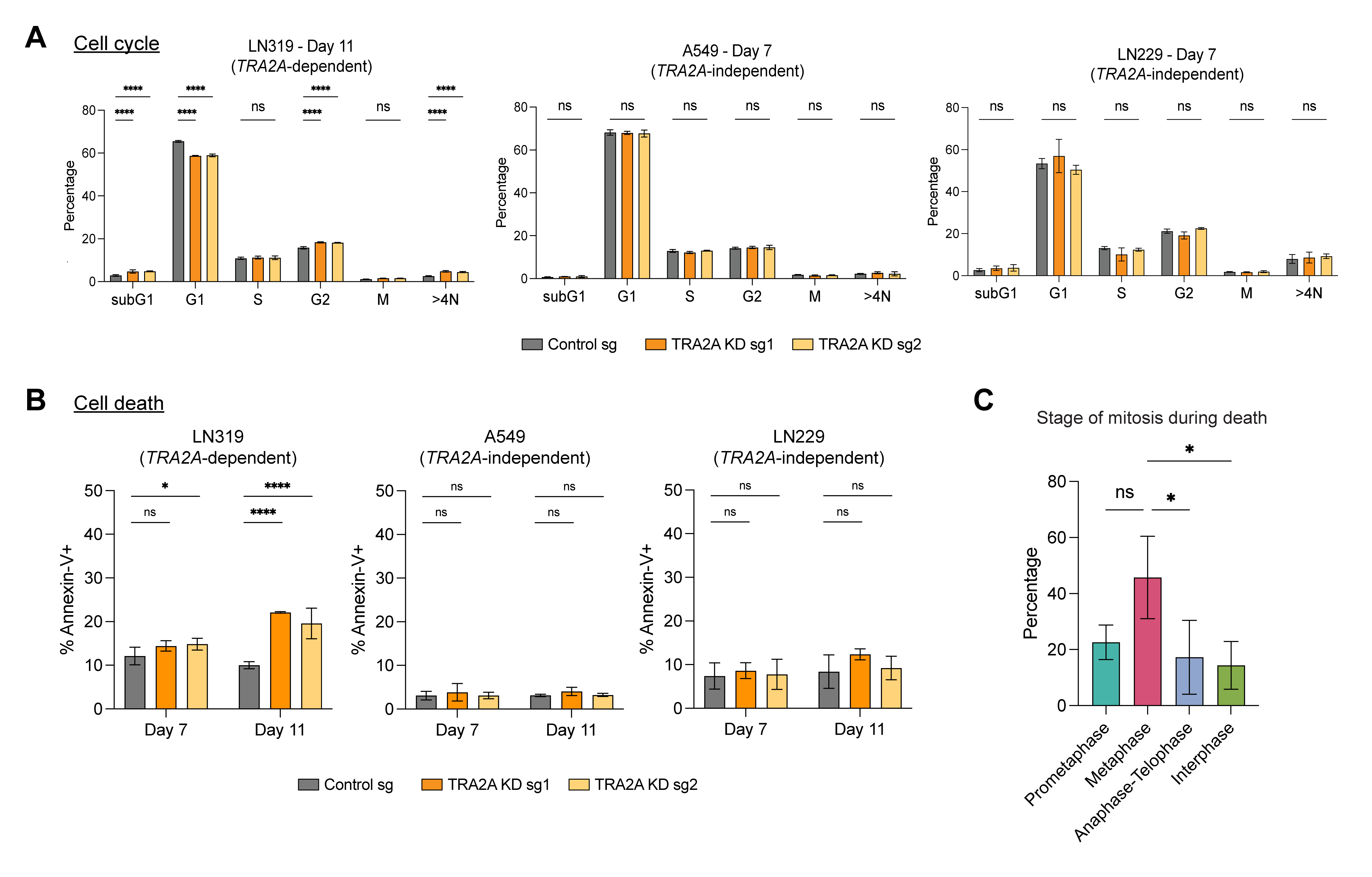

Supplement: S5 Fig — (A) Percentage of cells in each cell cycle stage upon control or TRA2A KD at Day 7 or 11 after infection in indicated cell lines, measured by propidium iodide and phospho-Histone H3 staining followed by flow cytometry, n = 3. (B) Percentage of apoptotic cells upon control or TRA2A KD at Day 7 or 11 after infection in indicated cell lines, measured by Annexin-V staining followed by flow cytometry, n = 3. (C) Percentage of dying mitotic cell population in each stage of mitosis during death in NCI-H23 cells, measured by live cell imaging, n = 3. Error bars represent standard deviation from the mean. (*)P < 0.05 and (ns) not significant, as calculated by repeated measures two-way ANOVA followed by Dunnett’s multiple comparison test. For (A,B) error bars represent standard deviation from the mean. (*)P < 0.05, (****)P < 0.0001, and (ns) not significant, as calculated by repeated measures two-way ANOVA followed by Dunnett’s multiple comparison test. (TIF) [file pgen.1011685.s005.tif]

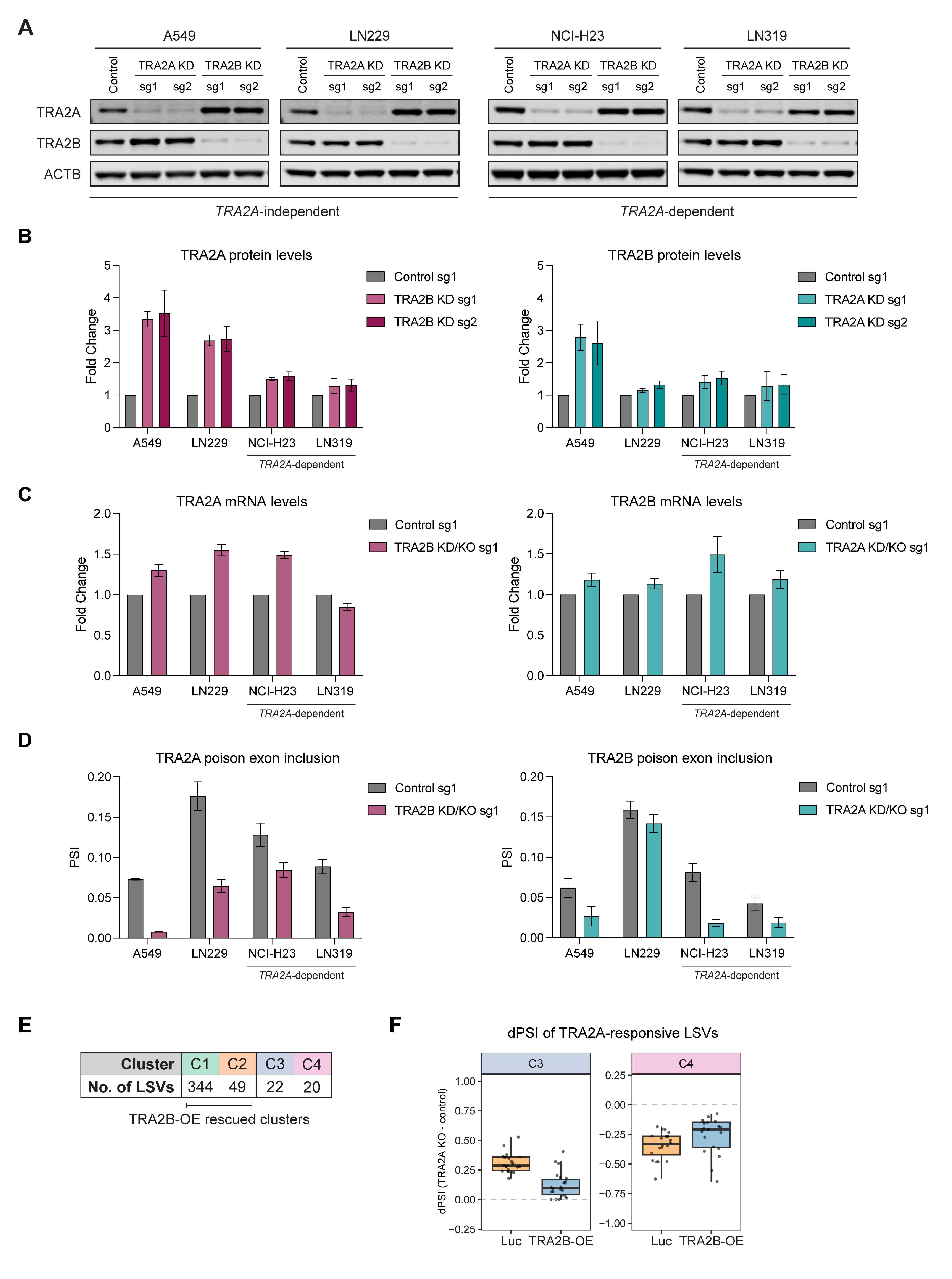

Supplement: S6 Fig — (A) Representative immunoblots measuring protein expression of TRA2A and TRA2B upon knockdown of TRA2A or TRA2B. ACTB was used as loading control. (B) Quantification of TRA2A protein upon TRA2B depletion (left) and TRA2B protein upon TRA2A depletion (right) relative to ACTB as in (A), n = 3. Error bars represent standard deviation from the mean. (C) RNA expression of TRA2A upon TRA2B depletion (left) and TRA2B upon TRA2A depletion (right) in indicated cell lines, as measured by RNA-seq. Error bars represent standard deviation from the mean. (D) Splicing inclusion of TRA2A poison exon upon TRA2B depletion (left) and TRA2B poison exon upon TRA2A depletion (right) in indicated cell lines, measured by RNA-seq and quantified by MAJIQ. Error bars represent standard deviation from the mean. (E) Number of LSVs in each cluster of the heatmap in Fig 6C. (F) Mean dPSI of TRA2A KO-responsive LSVs for clusters C3 and C4 in luciferase and TRA2B-OE conditions. (TIF) [file pgen.1011685.s006.tif]
